# Targeted Expression of Channelrhodopsin-2 to the Axon Initial Segment Alters the Temporal Firing Properties of Retinal Ganglion Cells

**DOI:** 10.1371/journal.pone.0142052

**Published:** 2015-11-04

**Authors:** Zhifei Zhang, Jie Feng, Chaowen Wu, Qi Lu, Zhuo-Hua Pan

**Affiliations:** 1 Department of Anatomy and Cell Biology, Wayne State University School of Medicine, Detroit, Michigan, United States of America; 2 Department of Physiology and Pathophysiology, School of Basic Medical Sciences, Xi’an Jiaotong University Health Science Center, Xi’an, China; 3 Department of Ophthalmology, Kresge Eye Institute, Wayne State University School of Medicine, Detroit, Michigan, United States of America; NIH/NEI, UNITED STATES

## Abstract

The axon initial segment (AIS) is essential for initiating action potentials and maintaining neuronal excitability in axon-bearing neurons in the CNS. There is increasing interest in the targeting of optogenetic tools to subcellular compartments, including the AIS, to gain precise control of neuronal activity for basic research and clinical applications. In particular, targeted expression of optogenetic tools in retinal ganglion cells (RGCs) has been explored as an approach for restoring vision after photoreceptor degeneration. Thus, understanding the effects of such targeting on spiking abilities and/or patterns is important. Here, we examined the effects of recombinant adeno-associated virus (rAAV)-mediated targeted expression of channelrhodopsin-2 (ChR2)-GFP with a Na_V_ channel motif in mouse RGCs. We found that this targeted expression disrupted Na_V_ channel clustering at the AIS and converted the spike firing patterns of RGCs from sustained to transient. Our results suggest that the clustering of membrane channels, including Na_V_ channels, at the AIS is important for the ability of RGCs to generate sustained spike firing. Additionally, the targeting of optogenetic tools to the AIS with the Na_V_ channel motif may offer a way to create transient light responses in RGCs for vision restoration.

## Introduction

In neurons, action potentials (APs) play a central role in cell-to-cell communication. Precisely patterned APs are required for neurons in the CNS to process and deliver complicated information. The axon initial segment (AIS) is known to be essential for initiating APs and maintaining neuronal excitability [1]. Recently, there is increasing interest in the targeting of optogenetic tools, such as channelrhodopsin-2 (ChR2), to subcellular compartments, including the AIS, to gain precise control of neuronal activity [2–4]. Thus, understanding the effects of such targeting on spike firing abilities and/or patterns is important.

Retinal ganglion cells (RGCs) are the final output neurons of the retina and fire APs that transmit visual information to higher visual centers in the brain. The ectopic expression of optogenetic tools in RGCs has been explored as a promising approach for restoring vision to the blind after retinal degeneration [5–7]. To this end, the restoration of intrinsic spatiotemporal coding of RGCs that mimics their native information processing could be important to achieve better outcomes of the restored vision.

One of the essential visual processing features of the retina is the segregation of sustained and transient visual signals, which convey distinct visual information, such as motion and edge vs. color and acuity, to the higher visual centers of the brain [8,9]. There are sustained and transient RGCs based on their temporal spiking properties to light; but ChR2-mediated light responses of non-targeted RGCs were predominantly sustained [5]. Thus, the ability to create ChR2-mediated transient light responses in RGCs is desired.

AISs are characterized by high densities of voltage-gated Na^+^ (Na_V_) and other membrane channels [10–12]. The clustering of these channels at the AIS is maintained by a complex of macromolecular proteins, among which Ankyrin-G (AnkG), a cytoskeletal linker protein, is the most important [13,14]. A conserved AnkG binding motif in the II-III cytoplasmic linker of all Na_V_1 subunits has been shown to be capable of directing proteins to the AIS [15,16]. In this study, we examined the effects of the expression of ChR2-GFP with this Na_V_ channel motif in RGCs. We found that this targeted expression disrupted the Na_V_ channel clustering at the AIS and converted the ChR2-mediated spike activities of RGCs from sustained to transient. Our results suggest that the clustering of membrane channels, including Na_V_ channels, at the AIS is important for the ability of RGCs to maintain sustained spike firing. Additionally, our findings may offer a way to create the transient light responses of RGCs with optogenetic tools for restoring vision.

## Materials and Methods

### DNA and Viral Vector Construction

ChR2-GFPs with or without the Na_V_ channel motif were delivered via a rAAV serotype 2/2 vector (rAAV2/2) [5]. The Na_V_ channel motif contained a 27-amino acid AnkG binding domain from the II-III cytoplasmic linker of the Na_V_1.6 channel (Na_V_II-III) that was fused in fame to the 3’ end of the GFP coding fragment [15,17]. Viral vectors with CAG promoter were packaged and affinity purified at the Gene Transfer Vector Core of the University of Iowa.

### Animals and Viral Vector Injection

All animal experiments and procedures were approved by the Institutional Animal Care and Use Committee (IACUC) of Wayne State University and were in accordance with the NIH *Guide for the Care and Use of Laboratory Animals*. Intravitreal viral injections were performed on adult C57BL/6J mice of either sex between 1 and 2 months of age according to previously described procedures [5]. The injections were performed in both eyes according to the protocol approved by the IACUC. Animals were anesthetized by intraperitoneal injection of a mixture of 120 mg/kg ketamine and 15 mg/kg xylazine. The concentrations of the control and motif-targeted vectors were 4.0 x 10^12^ GC/ml and 1.2 x 10^13^ GC/ml, respectively. The health of the animals was monitored daily by the staffs of the Division of Laboratory Animal Resources of Wayne State University. The virus injection was not expected to cause visual impairment of the animals. No animal death associated with the virus injection occurred. The animals were sacrificed for experiments at least one month after viral injection.

### Histology/Immunohistochemistry and Data Analyses

The animals were sacrificed by CO_2_ asphyxiation followed by decapitation. The retinas were fixed in the eyecups with 4% paraformaldehyde in 0.1 M phosphate buffer (PB; pH 7.4) for 20 min at room temperature (RT). The retinas were dissected in PB solution, flat mounted on slides, and coverslipped. For immunostaining, retinal whole-mounts were blocked for 45 min in a solution containing 5% ChemiBLOCKER (Millipore), 0.5% Triton X-100, and 0.05% sodium azide (Sigma-Aldrich, St. Louis, MO, USA). The primary antibodies were diluted in the same solution and applied for 24–72 hours, followed by incubation with the secondary antibodies, which were conjugated to Alex 594 (1:6000, red fluorescence) and Alexa 488 (1:600, green fluorescence; Thermo Fisher Scientific, Waltham, MA, USA). The primary antibodies were mouse anti-Ankyrin-G (1:10,000; Cat. 73–146; NeuroMab, Davis, CA, USA) and rabbit anti-pan-Na (1:1000; Cat. Asc-003; Alomone Labs, Jerusalem, Israel).

All images were made using a Zeiss Axioplan 2 microscope (Carl Zeiss, Oberkochen, Germany) with the Apotome oscillating grating to reduce out-of-focus stray light. Z-stack images were captured and displayed as maximum intensity projections. Further processing of the images was performed using ImageJ software (obtained from NIH).

The relationship between ChR2-GFP-NavII-III expression and pan-Nav immunostaining was analyzed in the merged z-stack images from retinal whole-mounts using ImageJ (obtained from NIH). Each image chosen for analysis contained multiple AISs. Each AIS with length ranging from 15 to 30 μm was defined based on showing either GFP or pan-Nav immunostaining or both. To measure GFP and pan-Nav fluorescence intensities (FIs), the fluorescence values of GFP and Nav in each defined AIS after background subtraction were divided by the number of pixels of the AIS. In each image, the measured FIs for GFP and pan-Na_V_ were normalized to the highest GFP FI and pan-Na_V_ FI, respectively. The relationships between the normalized pan-Nav FIs and GFP-FIs were plotted and analyzed with linear regression.

### Multi-Electrode Array Recordings

Multi-electrode array recordings were performed based on previously described procedures [5,18]. Briefly, the retina was dissected and placed photoreceptor-side down on a piece of nitrocellulose filter paper (Millipore Corp., Bedford, MA, USA). The mounted retina was placed in the MEA-60 multi-electrode array-recording chamber of 30 mm diameter with electrodes spaced 200 μm apart (Multi Channel System MCS GmbH, Reutlingen, Germany) with the ganglion cell layer facing the recording electrodes. The retina was continuously perfused in oxygenated extracellular solution at ~35°C. The extracellular solution contained (in mM): NaCl, 124; KCl, 2.5; CaCl_2_, 2; MgCl_2,_ 2; NaH_2_PO_4_, 1.25; NaHCO_3_, 26; and glucose, 22 (pH 7.35 with 95% O_2_ and 5% CO_2_). Photoreceptor-mediated light responses were blocked by the combination of 6-Cyano-7-nitroquinoxaline-2,3-dione (CNQX; 25 μM), D-(–)-2-Amino-5-phosphonopentanoic acid (D-APV; 25 μM), L-(+)-2-Amino-4-phosphonobutyric acid (L-APB; 10 μM). All the chemicals were purchased from Sigma-Aldrich (St. Louis, MO, USA). The interval between the onsets of each light stimulus was 20 s. The signals were filtered between 200 Hz (low cut off) and 20 kHz (high cut off) and recorded with MC Rack software (Multi Channel Systems). The responses of individual neurons were analyzed using Offline Sorter software (Plexon, Inc., Dallas, TX, USA). Spike raster plots and averaged spike rate histograms were generated using the NeuroExplorer software (Nex Technologies, Madison, AL, USA).

### Patch-Clamp Recordings

Patch-clamp recordings were performed from RGCs in retinal whole-mounts. The extracellular solution contained the following (in mM): 125 NaCl, 2.5 KCl, 1 MgSO_4_, 2 CaCl_2_, 1.25 NaH_2_PO_4_, 20 glucose, and 26 NaHCO_3_, bubbled with a gaseous mixture of 95% O_2_/5% CO_2_. Oxygenated medium was continuously perfused into the recording chamber at a rate of ~2–3 ml/min, and the retina was kept at ~34°C by a temperature-controller (TC-324B; Warner Instruments, Hamden, CT, USA). The electrode solution contained (in mM): 129 K-gluconate, 10 HEPES, 10 KCl, 4 Mg-ATP, and 0.3 Na-GTP with pH adjusted with KOH to 7.4. In voltage-clamp recordings, the membrane potential was held at –80 mV. In current-clamp recordings, the membrane potential was held around –80 mV, usually with a small negative holding current. The liquid junction potential was corrected.

### Light Stimulation

In whole-mount patch clamp recordings, light stimulation was generated by a 175 W xenon-based lamp (Lambda LS, Sutter Instrument, Novato, CA, USA) and coupled to the microscope through an optical fiber with a band-pass filter of 420–490 nm. For multichannel recordings, light stimulation was generated by fiber couple laser system at 473 nm (Changchun New Industries Optoelectronics, Changchun, China). The light intensity was attenuated by neutral density filters.

## Results

### rAAV-Mediated Na_V_II-III-Targeted Expression of ChR2-GFP Disrupt Na_V_ Channel Clustering at the AISs of RGCs

**Fig 1:**
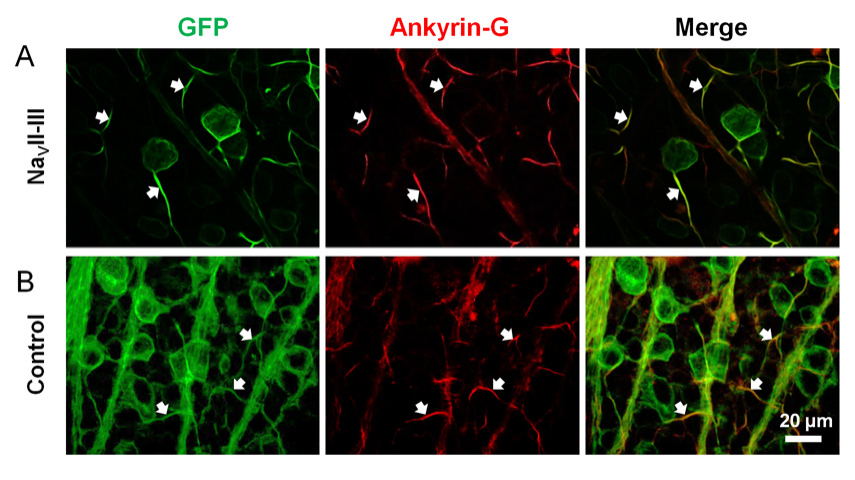
Immunohistochemical characterization of ChR2-GFP expression in RGCs with and without the Na_V_II-III-targeting motif. (A) In motif-targeted RGCs, the expression of ChR2-GFP was concentrated in the AISs and co-localized with the AIS marker AnkG (arrows). (B) In non-motif-targeted RGCs, the expression of ChR2-GFP was not concentrated in the AIS. The arrows point to the AnkG stained AIS.

The *In vivo* expression of ChR2-GFP with the Na_V_ channel motif Na_V_II-III, ChR2-GFP-Na_V_II-III, in RGCs was accomplished via the delivery of rAAV2/2 vectors by intravitreal injection. The expression was highly concentrated in the initial portion of the axons based on GFP fluorescence intensities (Fig 1A; left panel, arrows), which suggest that the expression was targeted to the AISs of RGCs. To confirm AIS-specific targeting, we used an anti-Ankyrin-G (AnkG) antibody that labels the AIS-specific protein AnkG (Fig 1A; middle panel, arrows). Indeed, the concentrated GFP fluorescence and AnkG staining were co-localized (Fig 1A; right panel). In contrast, the expression of ChR2-GFP-control (without the motif) was homogenous throughout the entire cell of the labeled RGCs including the AISs which exhibited concentrated AnkG staining (Fig 1B). These results confirm that rAAV-mediated expression of ChR2-GFP with the Na_V_II-III motif effectively targeted the ChR2-GFP to the AISs of RGCs.

**Fig 2:**
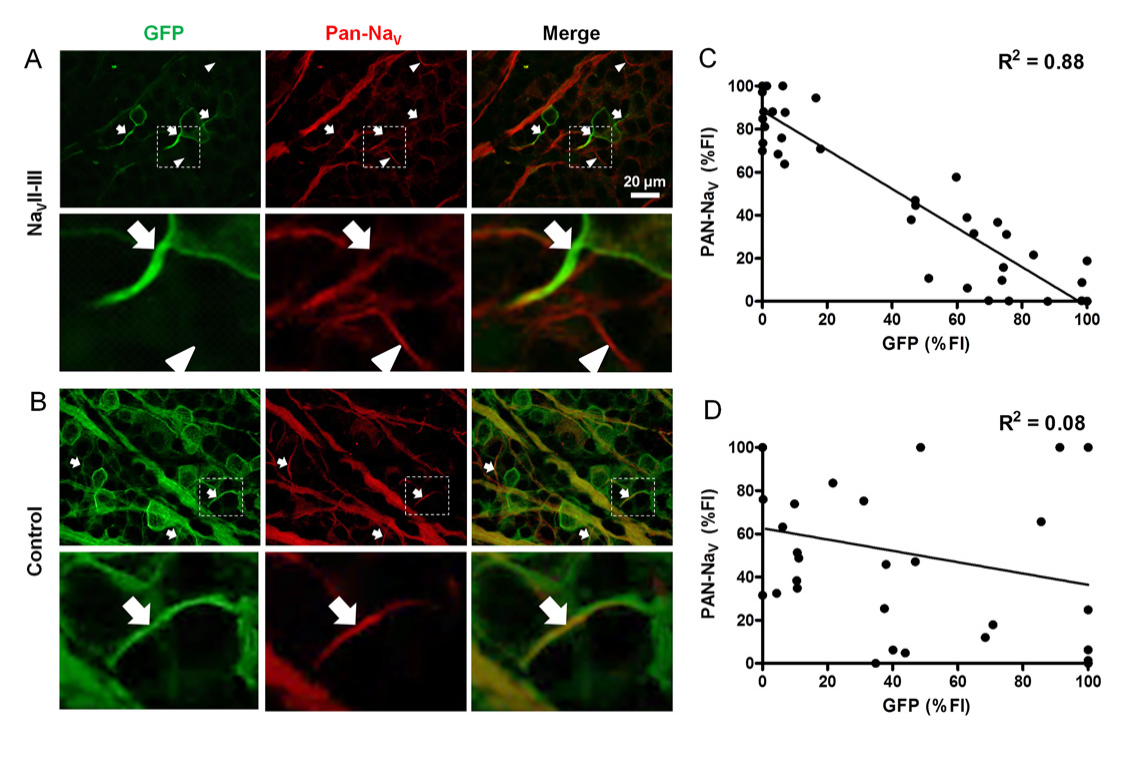
Relationship between ChR2-GFP expression and pan-Na_V_ immunostaining in the AISs of RGCs with the Na_V_II-III targeting motif. (A) Co-staining of ChR2-GFP and anti-pan-Na_V_ in motif-targeted RGCs. In the strongly ChR2-GFP-labeled AISs, co-staining with pan-Na_V_ was absent or weak (arrows). In the unlabeled or weakly ChR2-GFP-labeled AISs, co-staining with pan-Na_V_ was observed (arrowheads). Bottom panels are enlarged view of the boxed areas. (B) In non-motif-targeted RGCs, both ChR2-GFP expression and pan-Na_V_ staining were observed (arrows). Bottom panels are enlarged view of the boxed areas. (C) Scatter plot with linear regression line showing the inverse relationship between the normalized FIs of ChR2-GFP expression and pan-Na_V_ immunostaining in the AISs of RGCs with the targeting motif. (D) The scatter plot between the normalized FIs of ChR2-GFP expression and pan-Na_V_ immunostaining in the AISs of RGCs in the control group.

To determine whether the targeted expression of ChR2-GFP with the Na_V_II-III motif could compete with the binding of Na_V_ channels to AnkG [15,16], we used a pan-Na_V_ antibody to examine the spatial distributions of Na_V_ channels in the motif-targeted (Fig 2A) and control retinas (Fig 2B). Na_V_ channel staining in the AISs of the motif-targeted RGCs was varied in a manner that appeared to be related to the GFP fluorescence intensity. The pan-Na_V_ staining in areas of highly concentrated ChR2-GFP was minimal or absent (arrows in Fig 2A). In areas in which the expression of ChR2-GFP was absent, the pan-Na_V_ staining (arrowheads in Fig 2A) was comparable to that in the controls (arrows in Fig 2B). We quantitatively examined this effect by measuring the FIs of the pan-Na_V_ staining and GFP from each individual AISs of the motif-targeted RGCs (*n* = 42). We found an inverse relationship between the normalized pan-Na_V_-FI and the normalized GFP-FI (β = –0.91, F_(1, 40)_ = 283.2, R^2^ = 0.88, *p* <0.001; Fig 2C). In contrast, no such relationship was shown for the non-motif target group (*n* = 33; β = –0.19, F_(1, 31)_ = 113.2, R^2^ = 0.08, *p* = 0.267; Fig 2D). This result indicates that the expression of ChR2-GFP-Na_V_II-III disrupted Na_V_ channel clustering at the AIS, which suggests that the Na_V_II-III targeting motif competed against the endogenous Na_V_ channel for the AnkG binding site.

### The Targeted Expression of ChR2-GFP-Na_V_II-III Altered the Temporal Firing Patterns of RGCs

**Fig 3:**
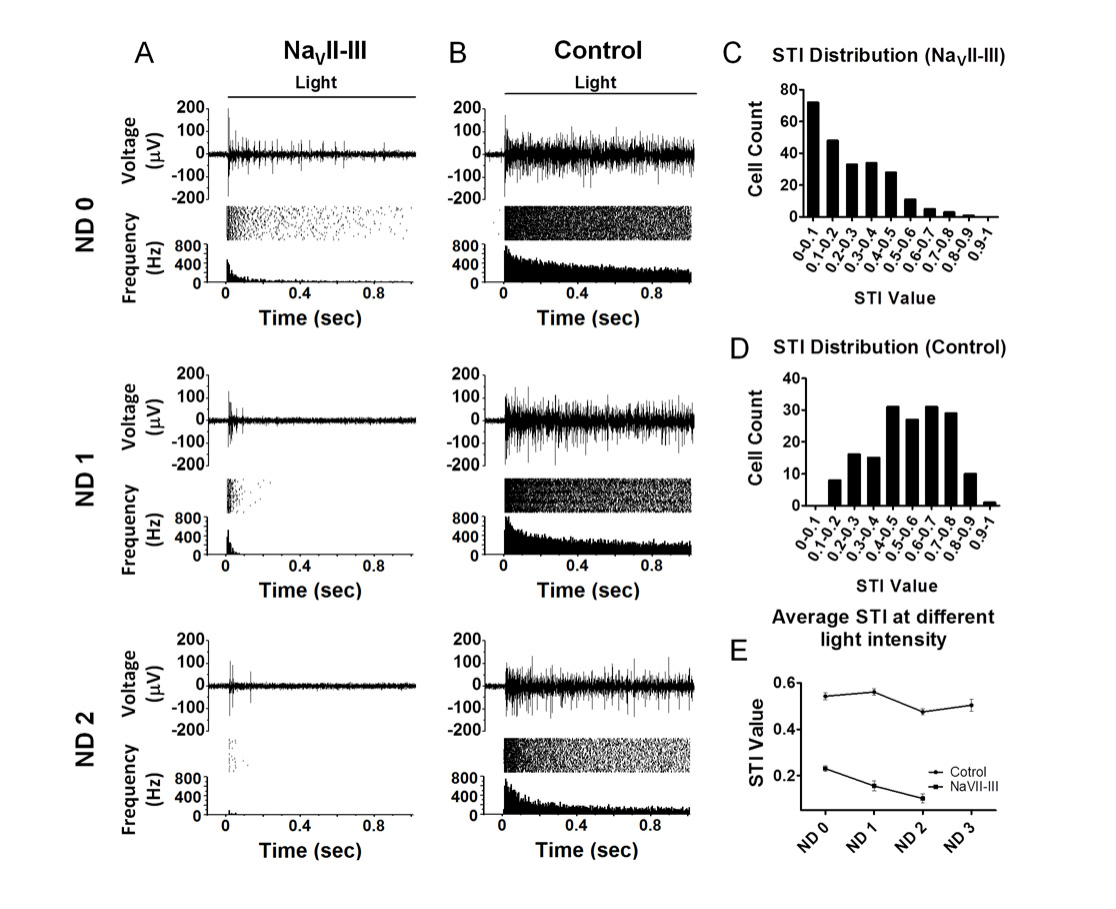
Comparison of ChR2-mediated spiking responses of motif-targeted and control RGCs in multi-electrode array recordings. Representative recordings of the ChR2-mediated spiking activities of the RGCs of motif-targeted (A) and ChR2-GFP-control (B) retinas. In each case, individual traces of light-evoked spike activity (upper panel), raster plots of 30 consecutive recordings (middle panel), and averaged spike rate histograms (lower panel) in response to three different light intensities (ND = 0, 1, 2 from top to bottom) are shown. The light intensity without neutral density filters (ND = 0) was 2.7 ×10^18^ photons cm^-2^s^-1^. (C) The STI distribution of the 237 motif-targeted RGCs. (D) The STI distribution of the 170 control RGCs. STI = spike counts in the 2nd 200 ms of light stimulation/spike counts in the 1st 200 ms of light stimulation. E) Average STI values under different light intensities for the motif-targeted (square; *n* = 40–235) and control (circle; *n* = 55–168) RGCs. The data are shown as the Mean ± SEM.

To examine the effect of the disruption of endogenous channel clustering at the AIS on the response properties of RGCs, we first assessed the ChR2-mediated spike firing properties of the RGCs via multi-electrode array recordings in whole-mount retinas. The photoreceptor-mediated light responses were blocked by CNQX (25 μM), D-APV (25 μM), and L-APB (10 μM). One-second light pulse elicited ChR2-mediated spiking activities at different light intensities, which were adjusted with neutral density (ND) filters. The ChR2-mediated spike firings of the motif-targeted retinas were found to be transient; the spike firings of the majority of cells were not maintained for the entire 1 s-light stimulation, particularly at the low light intensities (i.e., NDs of 1 or 2) (Fig 3A). This property contrasted markedly with the responses of the non-motif-targeted retinas; as previously reported [5], the non-motif-targeted retinas exhibited ChR2-mediated spike patterns that were sustained and, in the vast majority of the cells, maintained throughout the 1 s-light stimulation at all light intensities (Fig 3B). In addition, the maximal firing rates of the motif-targeted RGCs were also reduced compared to those of the non-motif-targeted RGCs.

To quantitatively measure this effect, we calculated a sustained/transient index (STI) by dividing the mean spike frequency during the second 200 ms of light stimulation by that during the first 200 ms of light stimulation [19]. Thus, STI values that are closer to zero would indicate cells with more transient spiking properties. The distributions of STI values for the motif-targeted (n = 237) and control group (n = 170) under the strongest light condition (ND = 0) are shown in Fig 3C and 3D, respectively. The distribution of the STI values of the motif-targeted group was markedly and significantly shifted toward smaller STI values compared to the distribution of the control group (chi square, *p* <0.0001). The average STI values under different light intensities for the two groups are shown in Fig 3E. In the motif-targeted group, the values were approximately 0.1–0.2 and decreased with reduction in light intensity; whereas, in the control group, the values were approximately 0.5–0.6 and constant across all light intensities. Thus, the targeted expression of ChR2 with the Na_V_II-III motif converted the ChR2-mediated spike firing patterns of RGCs from sustained to transient. Additionally, the activation thresholds of the motif-targeted retinas were higher than those of the control retinas as evidence by the finding that no spikes were evoked at the light intensity of ND = 2 in the motif-targeted group (Fig 3E).

### Patch-Clamp Characterization of RGCs Cells with ChR2-GFP-Na_V_II-III

**Fig 4:**
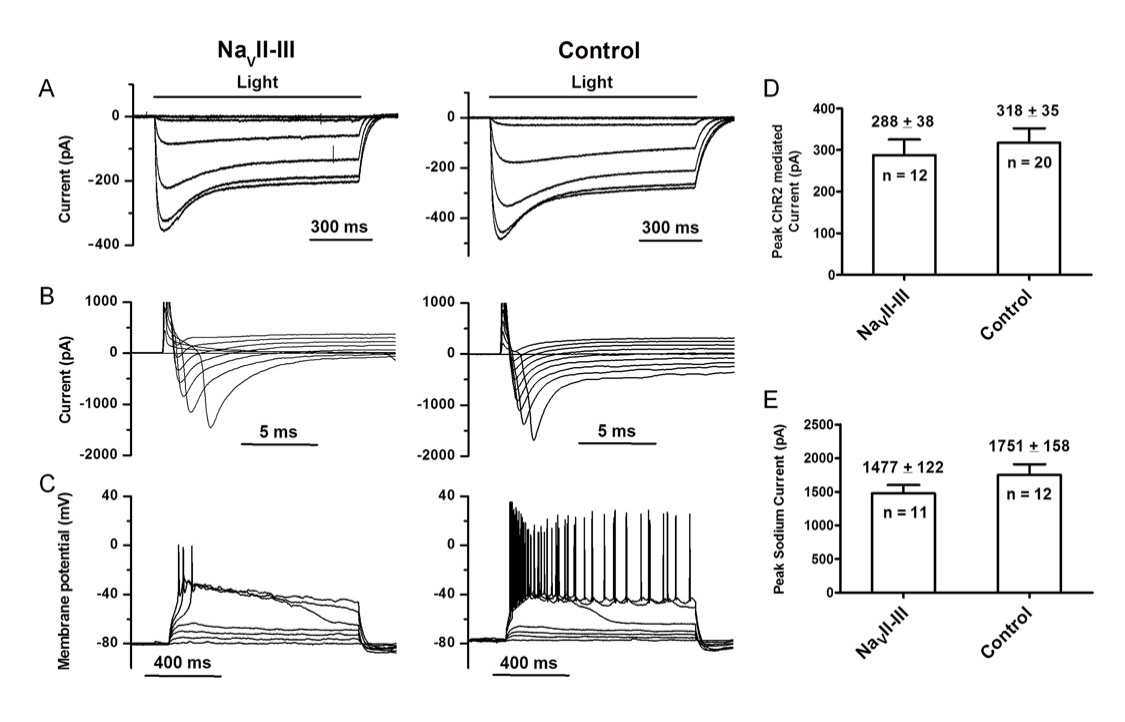
Comparison of ChR2-mediated currents, voltage-gated Na^+^ currents, and current-injection elicited spike responses between the motif-targeted and control RGCs. (A–C) Representative recording from the motif-targeted RGCs (left panel) and the control RGCs (right panel). The ChR2-mediated currents were evoked by incremental light intensities (ND 4.5 to 0) in voltage-clamp mode (A). Voltage-gated Na^+^ currents were evoked by voltage steps ranging –70 mV to +10 mV from the holding potential of –80 mV (B). Spike activities were elicited by 10-pA incremental current steps from 10 to 70 pA in current-clamp (C). (D) The average peak ChR2-mediated currents of the motif-targeted and control groups. (E) The average peak voltage-gated Na^+^ currents of the motif-targeted and control groups. The data are shown as the Mean ± SEM.

To investigate the underlying mechanism(s) of the alternation in spike firing pattern, we performed whole-cell patch-clamp recordings from RGCs in retinal whole-mounts. We first compared the ChR2-mediated currents between the motif-targeted and control groups in voltage-clamp mode. Again, the photoreceptor-mediated light responses were blocked by CNQX (25 μM), D-APV (25 μM), and L-APB (10 μM). Large ChR2-mediated currents were observed in both groups (Fig 4A). The amplitudes of the ChR2-mediated currents were not significantly different between the two groups (*t*-test, *p* >0.05; Fig 4D), which suggests that the targeted expression did not significantly alter the total numbers of expressed ChR2 channels in the RGCs. We also compared the voltage-gated Na^+^ currents between the two groups. The Na^+^ currents of the two groups were comparable (Fig 4B). The average peak Na^+^ currents of the two groups were also not statistically different (*t*-test, *p* >0.05; Fig 4E). The latter finding suggests that motif targeting did not significantly reduce the total numbers of Na_V_ channels in the RGCs. Finally, we examined the properties of current injection-elicited spike firing in current-clamp mode. Action potentials were observed in the majority of the cells of the targeted group (11 out of 12) in response to current injection, but only single spikes were observed (left panel in Fig 4C). In contrast, multiple spikes were observed in the majority of RGCs in the control group (14 out of 21). The majority of these cells (13 out of 14) spiked for the entire duration of the current injection (right panel in Fig 4C). Together, these results indicate that the motif-targeted RGCs lost the ability to generate sustained spike firing.

## Discussion

In this study, we showed that rAAV-mediated expression of ChR2-GFP with the Na_V_ channel-targeting motif Na_V_II-III was able to target the expression of ChR2-GFP to the AISs of RGCs. This targeted expression resulted in the disruption of Na_V_ channel clustering in the AISs. Moreover, the ChR2-mediated spike firing exhibited by the motif-targeted RGCs was markedly more transient than that of the control RGCs.

Our results suggest that this alteration of temporal firing properties was not due to a reduction in the total numbers of ChR2 or Na_V_ channels in the targeted RGCs because the magnitudes of the ChR2-mediated currents and voltage-gated Na_V_ currents were not significantly different between the control and motif-targeted groups. Rather, the machinery for generating sustained spike firing in the motif-targeted RGCs was impaired, as indicated by the finding that the motif targeted RGCs had lost the ability to generate sustained firing in response to current injection. The inability to generate sustained APs may be due to an insufficient Nav channel density at the AIS and/or that the mechanism of recovery from inactivation is compromised. Further studies are needed to understand the underlying mechanism.

The role of Na_V_ channels in the AIS in maintaining high spike firing rates in RGCs has previously been reported [20]. Na_V_ channels in the AISs of RGCs have been shown to contain both Na_V_1.6 and Na_V_1.1, which form micro-domain with Na_V_1.1 and Na_V_1.6 in the proximal and distal portions of the AIS, respectively [21]. The deletion of Na_V_ 1.6 in knockout mice has been shown to impair the maximal sustained and instantaneous firing rates of RGCs; however, the cells are still capable of generating sustained firing, albeit at a lower frequency. The retention of this sustained firing ability is possibly due to the expression of the remaining Na_V_1.1 and/or the compensatory expression of Na_V_1.2 at the AIS [20]. These previous studies indicate that both the intrinsic properties and the locations of Na_V_ channels are important for the generation of precise AP firing patterns. In the current study, since the AnkG-binding site for Na_V_II-III is conserved among all Na_V_ channel subtypes [15,16], the Na_V_II-III targeting motif would disrupt the clustering for all Nav channels at the AIS. Although the mechanism of how the expression of the targeted ChR2 results in the disruption of Nav channel clustering at the AIS remains to be investigated, our results are consistent with a role of Na_V_ channel clustering in maintain sustained spike firing. In addition, since other membrane channels such as certain voltage-dependent K^+^ channels are also known to be clustered at the AIS through binding to AnkG [10,11,22,23], the Na_V_II-III motif targeting may also disrupt other membrane channels at the AIS, which may contribute to the effect. Nevertheless, our results suggest that the clustering of membrane channels, including Na_V_ channels, at the AIS is essential for the ability of RGCs to generate sustained spike firing.

Interestingly, a recent study reported that targeted expression of ChR2 with a Na_V_ channel motif in cultured hippocampus neurons resulted in minimal ChR2-mediated currents that failed to elicit any action potentials under normal conditions [4]. This study also reported that normal spike firing could be elicited by current injection, which suggests that the spike firing machinery of these targeted hippocampus neurons was intact. Notably, the motif sequences used in this previous and ours were different: a short sequence of 27 amino acids was used in the current study, whereas the entire II-III cytoplasmic sequence of 228 amino acids was used in the study of cultured hippocampal neurons. Because Na_V_ channel clustering at the AIS in the targeted hippocampal neurons was not examined, it is unknown whether the discrepancy between these two studies is due to differences in the Na_V_ channel motifs or the types of the neurons examined.

The results of this study have a potential clinical application for the optogenetic approaches to restoring light sensitivity at the level of RGCs [5–7]. For improving the outcome of the restored vision, the restoration of the intrinsic information processing of RGCs, such as ON/OFF by using depolarizing and hyperpolarizing optogenetic tools and center-surround receptive fields by using center- and surround-targeting motifs, has been sought by previous studies [19,24,25]. The segregation of sustained and transient visual signals is another important visual processing feature in the retina. RGCs are classified as sustained and transient cells based on their temporal spiking properties in response to prolong light stimuli. The sustained and transient responses are originated at the level of bipolar cells [26]. Consistently, the ChR2-mediated light responses by non-targeted expression of ChR2 in RGCs were predominantly sustained [5]. Similar finding was also reported by non-targeted expression of melanopsin in RGCs [7]. The conversion of ChR2-mediated spike firing from sustained to transient in the Na_V_ channel motif-targeted RGCs revealed in this study provides a possible strategy to recreate transient light responses in RGCs. As shown in this study, the expression of optogenetic tools, such as ChR2, with Na_V_ channel motifs can be used to create transient light responses. The creation of transient responses may also be achieved by altering channel clustering at the AIS via targeting based on other AIS targeting motifs. Additionally, the combination of the Na_V_ channel motif with the previously reported center- and surround-targeting motifs [24,25] could be used to create transient center or transient surround light responses. Furthermore, once RGC subclass-specific promoters become available, optogenetic tools with the targeting motifs could be targeted to specific subclass of RGCs for generating light responses to mimic their intrinsic spatial and temporal light response properties.
